# *Anopheles gambiae* APL1 Is a Family of Variable LRR Proteins Required for Rel1-Mediated Protection from the Malaria Parasite, *Plasmodium berghei*

**DOI:** 10.1371/journal.pone.0003672

**Published:** 2008-11-07

**Authors:** Michelle M. Riehle, Jiannong Xu, Brian P. Lazzaro, Susan M. Rottschaefer, Boubacar Coulibaly, Madjou Sacko, Oumou Niare, Isabelle Morlais, Sekou F. Traore, Kenneth D. Vernick

**Affiliations:** 1 Department of Microbiology, University of Minnesota, Saint Paul, Minnesota, United States of America; 2 Department of Entomology, Cornell University, Ithaca, New York, United States of America; 3 Malaria Research and Training Center, University of Bamako, Bamako, Mali; 4 Laboratoire de Recherche sur le Paludisme, Institut de recherche pour le développement IRD-OCEAC, Yaoundé, Cameroun; 5 Unit of Insect Vector Genetics and Genomics, Department of Parasitology and Mycology, CNRS Unit URA3012: Hosts, Vectors and Infectious Agents, Institut Pasteur, Paris, France; Indiana University, United States of America

## Abstract

**Background:**

We previously identified by genetic mapping an *Anopheles gambiae* chromosome region with strong influence over the outcome of malaria parasite infection in nature. Candidate gene studies in the genetic interval, including functional tests using the rodent malaria parasite *Plasmodium berghei*, identified a novel leucine-rich repeat gene, *APL1*, with functional activity against *P. berghei*.

**Principal Findings:**

Manual reannotation now reveals *APL1* to be a family of at least 3 independently transcribed genes, *APL1A*, *APL1B*, and *APL1C*. Functional dissection indicates that among the three known *APL1* family members, *APL1C* alone is responsible for host defense against *P. berghei*. APL1C functions within the Rel1-Cactus immune signaling pathway, which regulates *APL1C* transcript and protein abundance. Gene silencing of *APL1C* completely abolishes Rel1-mediated host protection against *P. berghei*, and thus the presence of APL1C is required for this protection. Further highlighting the influence of this chromosome region, allelic haplotypes at the APL1 locus are genetically associated with and have high explanatory power for the success or failure of *P. berghei* parasite infection.

**Conclusions:**

*APL1C* functions as a required transducer of Rel1-dependent immune signal(s) to efficiently protect mosquitoes from *P. berghei* infection, and allelic genetic haplotypes of the *APL1* locus display distinct levels of susceptibility and resistance to *P. berghei*.

## Introduction

Malaria is a global health problem resulting in over 1 million deaths annually, with disproportionate mortality in African children under the age of five [1]. Malaria also imposes a large economic burden on developing countries. Current efforts to control this disease are multifaceted and include use of insecticides and insect barriers, drug therapy, and strengthening healthcare and research infrastructures [2]. More consistent and widespread implementation of existing tools would be beneficial, although technical problems such as selection for chemico-resistance in vectors and parasites emphasize the need for a new generation of malaria control tools [3].

One such new approach could be limiting the genetic propensity of vector mosquitoes to serve as competent hosts for parasite development, thus decreasing or abolishing their ability to transmit the causative agent. This approach is in its infancy and much remains to be done before we can evaluate specific genetic resistance mechanisms and the feasibility of manipulating them in nature.

We designed a phenotype-based method to genetically screen the wild *A. gambiae* population for genomic regions important in defense against *P. falciparum* [4]. Using this approach, we identified a genetic locus on chromosome 2L that consistently explains >80% of the variation in infection outcome (i.e., surviving oocyst numbers) in mosquitoes exposed to an infective bloodmeal, and thus captures most of the natural genetic variation for *P. falciparum* resistance or susceptibility [5]. The genetic interval, currently ∼10 Mb, was termed the *Plasmodium*-Resistance Island (PRI). We then employed the rodent malaria laboratory model of *P. berghei* [6]–[12] to functionally screen candidate genes in the PRI. This work identified *APL1*, a novel leucine-rich repeat (LRR) containing protein [5]. When *APL1* transcript abundance was reduced by RNAi gene knockdowns, the number of *P. berghei* oocysts was increased up to 20-fold, showing it to be a potent factor for host defense against *P. berghei* infection [5].

Here, we reannotate the original *APL1* gene as a gene family of 3 related members, *APL1A*, *B*, and *C*. Gene-specific RNAi assays show that all of the malaria-protective activity we previously reported for *A. gambiae APL1* can now be attributed exclusively to *APL1C*. We functionally dissect the position of *APL1C* in mosquito immune signaling networks, placing *APL1C* as a required node in Rel1-mediated host defense against *P. berghei* infection. Finally, we identify haplotypes in the APL1 locus that are genetically associated with the degree of phenotypic susceptibility to *P. berghei* infection.

## Results

### Reannotation of the ENSEMBL prediction for *APL1*

**Figure 1:**
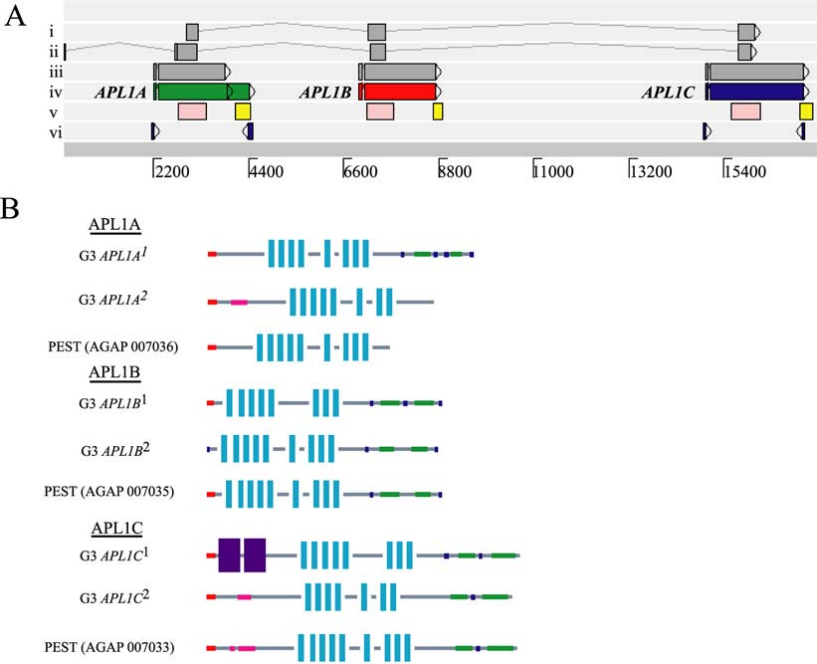
A. Reannotation of the *APL1* region. i) Ensembl release version 36, ii) Ensembl release version 41, iii) Ensembl release version 45, iv) Empirical annotation of *APL1A*, *B* and *C* in this article and Vectorbase manual annotation database, v) Fragments used for RNA interference assays; common dsRNA fragment knocking down *APL1A*, *B* and *C* (pink), unique dsRNA fragments at the 3′ end of each gene used for gene-specific knockdowns (yellow), vi) 5′ and 3′ RACE fragments used to delimit transcripts. B. APL1 family protein functional motifs. Predicted peptide domains are indicated as follows: red, signal peptide; green, coiled-coil domain; light blue vertical bars, leucine rich repeats; blue, regions of intrinsic disorder, pink, segments of low complexity; and purple, repeat regions. Haplotypic versions of the APL1 proteins as discussed in the text differ in functional predictions, with PEST strain predictions being most similar to the APL1A^2^, B^2^, and C^2^ haplotypic forms.

Examination of *APL1* at the time of our original description [5] suggested that its annotation as a single gene (ENSANGG00000012041 in ENSEMBL version 44 and earlier) was incorrect. The previous ENSEMBL prediction for *APL1* lacked start and stop codons, predicting a partial protein consisting of little more than a string of LRR domains. Resequencing of genomic DNA and archived clones from the original *A. gambiae* sequencing project [13], as well as transcript mapping, revealed that the previous *APL1* gene represented the erroneous annotation of a gene family comprised of at least 3 tandem LRR-containing genes, here named *APL1A*, *APL1B*, and *APL1C* (Figure 1A). Each of the 3 genes has a short 5′ exon followed by a small intron and a longer second exon, and each has a block of LRR motifs flanked by an N-terminal signal peptide and C-terminal coiled coil domains (Figure 1B). The three individual *APL1* genes display sequence similarity that likely results from gene duplication and functional diversification (diagonals in Figure S1), and we therefore class them together as the *APL1* family.

There were notable structural differences between the resequencing results and the ENSEMBL genome assembly (discussed in order below): i) the presence of structurally polymorphic haplotypes, and ii) a polymorphic and/or active transposable element. First, all three *APL1* family genes display major structural haplotypes (Figure 1B). The differences between allelic forms are most striking for *APL1A* and *APL1C*. Variants can differ in the locations of their predicted stop codons, resulting in predicted proteins of distinct lengths, and also by the presence of multiple polymorphic insertion-deletion (indel) sites within the protein coding sequence (CDS). The indels are precisely in-frame with the surrounding protein. Thus, the indels do not introduce missense mutations but rather encode small peptide cassettes that are present or absent, respectively, in the predicted finished protein. In a sample of wild and colony mosquitoes, indel alleles appear consistently linked to specific surrounding nucleotide variants (discussed below), thus establishing the indels as reliable markers for stable haplotypes that encode predicted proteins of distinct sizes and structure. The haplotypes are designated by the gene name followed by a superscript number (Figure 1B). The superscript 2 haplotype for each gene is most similar to the variant found in the PEST strain used for the *A. gambiae* genome sequence.

Another structural difference revealed by resequencing occurs upstream of the *APL1A* gene, where we found that a tract of Ns in the public genome assembly is actually (in PEST strain plasmid clone 19600445759751) a TA-III-Ag miniature inverted transposable element (MITE, [14]). The ambiguous bases in the ENSEMBL genome sequence may result from the difficulty in sequencing the repetitive region, or perhaps from the polymorphic state of the MITE in the sequence template. The G3 strain lacks the MITE in this genomic location.

### *APL1C* is required for the control of *P. berghei* infection

**Figure 2:**
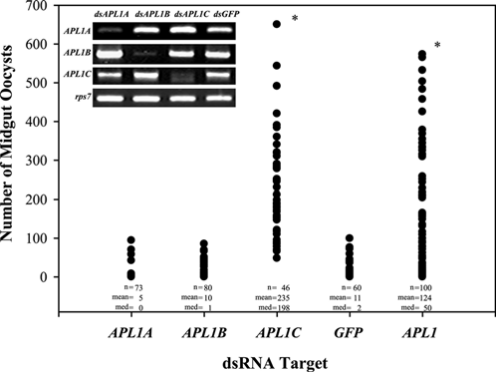
Among the *APL1* family, only *APL1C* specifically protects against *P. berghei* infection. The three *APL1* family genes were individually assayed to determine their relative contributions to host defense against *P. berghei* infection. Horizontal axis indicates the gene target of RNAi knockdown, where *APL1A*, *B* and *C* are gene-specific knockdowns, *GFP* is an irrelevant control dsRNA, and *APL1* is a shared dsRNA that simultaneously knocks down all 3 *APL1* family genes. Vertical axis indicates the number of midgut oocysts 7–8 d following IBM, with sample size (n), mean (mean), and median (med). Infection levels in *APL1A* and *APL1B* silenced mosquitoes were not different from *GFP* controls. However, treatment with either *dsAPL1C* or *dsAPL1* (targeting all 3 genes) permitted significantly greater oocyst development than the other treatments (asterisk, p<0.05 by Dunn's Multiple Comparison after Kruskal Wallis one way ANOVA on ranks). Infections of *APL1C* and *APL1* silenced mosquitoes were not different from each other, indicating that the function of *APL1C* alone is sufficient to explain all of the increased permissiveness caused by complete *APL1* family knockdown. The result for each knockdown target represents pooled data from at least 2 independent replicate experiments. One of the common *APL1* knockdowns was done alongside an *APL1C* kd, and when assayed in the same replicate, there was no significant difference between *APL1C* kd and common *APL1* kd (p = 0.481). The inset gel photo shows representative examples of gene knockdown efficiency for the 3 members of the *APL1* gene family. Labels above gel images indicate the dsRNA that was used for the knockdown. Labels to the left of images indicate the transcript detected by RT-PCR on RNA purified from the treated mosquitoes. *rps7* was used as a control for cDNA input in PCR.

We previously demonstrated that the *APL1* family had a pronounced effect on *P. berghei* oocyst intensity in an RNAi gene expression knockdown assay [5]. In that case, the double-stranded RNA (dsRNA) fragment injected into mosquitoes was fortuitously common to a portion of all three *APL1* family genes (Figure 1A, track v, homology regions of *APL1*-common dsRNA indicated by pink bars; also Figure S1B), because at the time *APL1* was annotated as a single gene. Based on our current reannotation of *APL1*, we wondered whether the source of our previously reported *APL1* knockdown phenotype was a single *APL1* family member, or alternatively a combined effect of all 3 members. To test this, we conducted knockdown experiments using new dsRNA constructs specific for each of the *APL1* family members (locations of dsRNAs shown in Figure 1A, track v, yellow bars). The results indicate that among the *APL1* family, *APL1C* alone is responsible for the control of *P. berghei* oocyst intensity (Figure 2). Oocyst loads in either *APL1A* or *APL1B* knockdown mosquitoes were statistically indistinguishable from those of the GFP controls, while *APL1C* knockdown mosquitoes carried oocysts loads ∼20 times greater than GFP controls (p<0.05). The effect of the *APL1*-common dsRNA fragment that silences the three genes was not different from the *APL1C* specific dsRNA, indicating that the effect of *APL1C* is both necessary and sufficient to explain APL1 protective function against infection with *P. berghei*.

### *APL1C* activity is required for anti-*P. berghei* protection mediated by the Toll/Rel1 pathway

The Toll signaling pathway controls cellular and humoral innate immune signaling, including activation of anti-fungal and anti-Gram positive bacteria defense in *Drosophila* [15], [16] The genes for the core components of the pathway are also found in the *A. gambiae* and *Aedes aegypti* genomes [17], [18]. Mosquito Rel1, ortholog of *Drosophila* Dorsal and functional analog of *Drosophila* Dif, is an ultimate transcription factor of the Toll pathway [11], [19], [20]. Under naïve conditions, Rel1 is retained in the cytoplasm due to binding of the inhibitor, Cactus. Activation of the Toll receptor by its ligand spatzle in response to pathogens results in the disassociation of Cactus from Rel1 [21]. Released Rel1 translocates to the nucleus where it transactivates target genes. Recently, Frolet et al. [11] have shown that Rel1 regulates the transcription of *TEP1* and *LRIM1*, two anti-*Plasmodium* genes in *A. gambiae*, and that depletion of the Rel1 inhibitor, Cactus, by RNAi strongly promoted mosquito host defense against *P. berghei*. In the same study, it was claimed that *APL1* was not transcriptionally regulated by Rel1. However, the PCR assay used for the *APL1* expression in their study was actually specific for the AGAP007037 gene (upstream of *APL1A*), which was incorrectly annotated as an exon of *APL1*.

**Figure 3:**
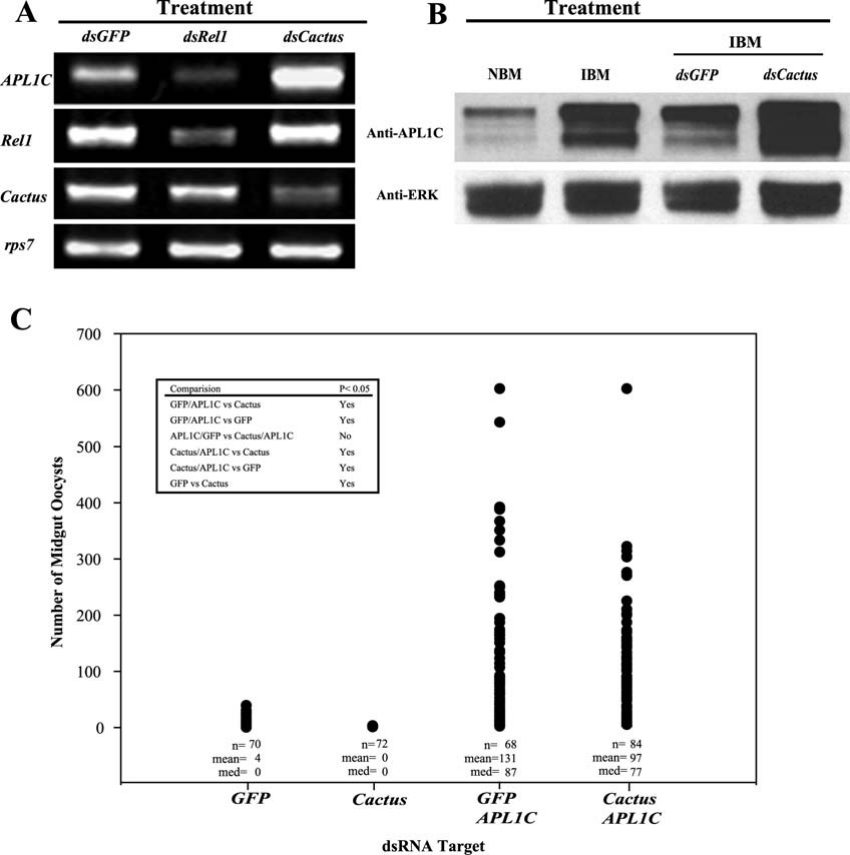
A. *APL1C* mRNA is regulated by the Rel1/Cactus immune signaling pathway. Semi-quantitative RT-PCR was used to measure the effect of *Rel1* and *Cactus* knockdown on *APL1C* transcript abundance. Labels above gel indicate the dsRNA that was used for the knockdown, labels to the left indicate the transcript whose abundance was measured. *APL1C* level was decreased by *Rel1* silencing, and increased by *Cactus* silencing, consistent with a model whereby *APL1C* RNA is regulated positively by Rel1 and negatively by Cactus. Specificity is shown by *dsRel1* and *dsCactus* silencing of cognate RNA levels. B. APL1C protein abundance is regulated by *P. berghei* infection and Cactus. Western blot analysis of APL1C protein abundance 24 h after *P. berghei* infection in naïve, *dsCactus* knockdown, and *dsGFP* control mosquitoes. Total protein abundance of ERK detected by anti-ERK antibodies was used as a protein loading control. C. APL1C is required for Rel1-mediated host-defense against P. berghei. The functional effect of *Cactus* and *APL1C* activity on the outcome of *P. berghei* infection was tested using gene knockdowns. The RNAi knockdown target is shown on the horizontal axis. *GFP* was used as a control dsRNA. The vertical axis shows the number of midgut oocysts 7–8 d following a P. berghei IBM. Sample sizes (n), means (mean), and medians (med) are given for knockdowns, which were each pooled data from 2 independent experiments. The oocyst loads differed significantly (p<0.001) among samples by Kruskal Wallis one way ANOVA on Ranks. Pairwise comparisons using Dunn's method of multiple comparisons are given in the inset box. ds*Cactus*-treated mosquitoes were completely protected from infection, with no successful oocyst development. In distinction, the double knockdown of *APL1C* simultaneously with *Cactus* produced mosquitoes with ∼25-fold more oocysts than the dsGFP-treated controls, a result that was no different than the ds*APL1C*-treated mosquitoes. Thus, in the absence of APL1C, Rel1 activation has no effect on parasite development, indicating the requirement of the presence of APL1C for Rel1-mediated host defense against *P. berghei*. Note that ds*GFP* was included in the *APL1C* knockdown so that total amount of input dsRNA per mosquito was the same as in the *Cactus-APL1C* double knockdown, eliminating dsRNA concentration as a variable.

A specific assay for the expression of *APL1C*, the only anti-*P. berghei* gene among the 3 *APL1* genes, reveals that *APL1C* is in fact regulated by Rel1, as determined by its expression in Rel1 and Cactus knockdown mosquitoes (Figure 3). *APL1C* transcription was reduced in ds*Rel1* treated mosquitoes, and increased in ds*Cactus* treated mosquitoes (Figure 3A). Furthermore, APL1C protein abundance was elevated following *P. berghei* infection, and this elevation was enhanced by ds*Cactus* (Figure 3B). Boosting Rel1 signaling by depletion of Cactus enables mosquitoes to eliminate almost all invaded malaria parasites within 48 h post-infection [11]. If *APL1C* is a host-protective gene regulated by Rel1/Cactus signaling, then the anti-*P. berghei* effect of ds*Cactus* should be reduced in the absence of *APL1C* expression. To test this hypothesis, we examined the effect of a ds*Cactus*/ds*APL1C* double knockdown on the outcome of *P. berghei* infection. As expected, control mosquitoes treated only with *dsCactus* eliminated all invaded parasites by either lysis or melanization responses. Most ds*Cactus* mosquitoes (24/36) harbored no parasites on day 7 post-IBM, while the remaining third of the mosquitoes (12/36) displayed melanized parasites (range 1–19 melanized parasites/mosquito) but no normal oocysts. Interestingly, this elevated parasite resistance of ds*Cactus* treated mosquitoes was completely reversed when *APL1C* was silenced simultaneously with *Cactus*, because the oocyst load in mosquitoes treated with ds*Cactus/*ds*APL1C* was equivalent to that in ds*APL1C*/ds*GFP* treated mosquitoes (Figure 3C). These results indicate that *APL1C* is a required mediator of the anti-*P. berghei* effect controlled by Rel1 signaling, because constitutive activation of Rel1 signaling by Cactus depletion, which is normally entirely protective for mosquitoes, had no effect in the absence of *APL1C*. Treatment of mosquitoes with dsRNA for two other mosquito immune factors, TEP1 and LRIM1, only partially reversed the dsCactus phenotype in double knockdowns, allowing development of some normal *P. berghei* parasites [11]. However, because Cactus depletion confers no host-protective phenotype without the presence of *APL1C*, it appears that the effect of *APL1C* is functionally dominant in this anti-*P. berghei* pathway. We hypothesize that *APL1C* may be a functionally upstream signaling node responsible for the coordinated Rel1-dependent control of multiple host protective factors.

### Haplotypes at the *APL1* locus are genetically associated with protection from *P. berghei* infection

Resequencing and reannotation of the *APL1* locus identified major structural haplotypes that include alleles of *APL1A*, *APL1B*, and *APL1C* (Figure 1B). We hypothesized that the genetic and predicted protein variation in this locus could control differences for host defense against *P. berghei*. If true, one could test for the phenotypic effect of different haplotypes by detecting association of haplotype-specific markers with phenotypic outcome after *P. berghei* infection.

**Figure 4:**
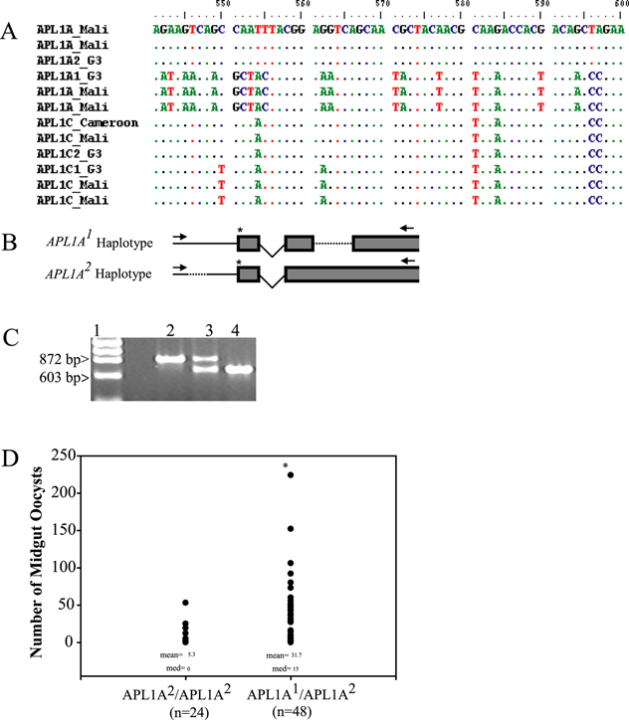
A. *APL1* alleles are comprised of blocks of linked SNPs. Nucleotide alignment shows *APL1A* and *APL1C* genetic variation in a sample of *A. gambiae* wild and colony mosquitoes. Haplotypes are shared over the resequenced region between individual mosquitoes, indicating that the variants are stable haplotypes that exist in *A. gambiae* at appreciable frequency. B. Genotyping assay for segregating indels in the *APL1 locus*. A PCR assay was designed to test the state of 2 indels in *APL1A* that are markers for allelic haplotypes at the *APL1* locus. Arrows show forward and reverse primers used in the diagnostic assay, dashed lines indicate indels (located within exon 2 of *APL1A^1^*, and above the translation start in *APL1A^2^*), asterisk indicates the start codon of *APL1A*. C. Indel genotyping assay. A representative gel of PCR products from the indel diagnostic assay, lane 1, Lambda HindIII/PhiX HaeIII marker, lane 2, *APL1A^2^* homozygote (854 bp), lane 3, a heterozygote with codominant bands, lane 4, *APL1A^1^* homozygote (663 bp). D. *APL1 locus* haplotypes control distinct levels of protection from *P. berghei* infection. Homozygous *APL1A^2^* mosquitoes were significantly less permissive for parasite development than *APL1A^1^*/*APL1A^2^* heterozygotes (asterisk, p<0.001, Mann Whitney Rank Sum Test; sample size (n), mean (mean), and median (med) given for each haplotype). Homozygous *APL1A^1^* individuals are present at low frequency in the sampled colony and thus were not included in the analysis (see Results for further details on *APL1A^1^* haplotype frequency).

To design a genetic assay for haplotype, we resequenced part of the *APL1A* and *APL1C* genes in a sample of colony and wild mosquitoes. Patterns of specific SNPs distinguish the *APL1* haplotypes and are stable among mosquitoes of distinct geographic origins (Figure 4A). Thus, we designed a simple PCR fragment length assay to detect the genotype of indels in *APL1A* (Figure 4B, C). Typing of multiple mosquitoes using the PCR assay indicated that the indels are linked to and diagnostic for the *APL1* haplotypes (not shown). In summary, the *APL1A^1^* haplotype bears the deleted variant of an indel located in exon 2, while *APL1A^2^* bears the inserted variant for this indel. *APL1A^2^* bears the deleted variant of an indel located 5′ to the predicted translation start site, while *APL1A^1^* bears the inserted variant of this indel (Figure 4B). The exon 2 indel is an in-frame CDS deletion, which consequently alters the length of the predicted protein product but does not introduce missense/nonsense mutations (not shown).

We challenged the G3 strain *A. gambiae* with a *P. berghei*-infected bloodmeal, and used the indel genotyping assay to query for an association between *APL1* genotype and infection outcome (Figure 4D). Mosquitoes that were homozygous for *APL1A^2^* had significantly lower oocyst loads (average = 5.3±2.5) than *APL1A^1^*/*APL1A^2^* heterozygotes (average = 31.7±6; Mann Whitney Rank Sum Test, p<0.001). Furthermore, the prevalence of infection in homozygous *APL1A^2^* mosquitoes was lower (37.5%; 9/24) than that in *APL1A^1^*/*APL1A^2^* heterozygotes (84.5%; 41/48; Fisher's Exact Test, p<0.001).

Thus, the haplotype-tagging indels are linked to genetic variation that controls significant difference in numbers of surviving *P. berghei* oocysts. We emphasize that the indels serve as genetic markers to detect the phenotypic effect of linked variant sequences, and linkage does not imply that the marker itself underlies the observed phenotypic variation. Like any other genetic marker in common use, for example almost all microsatellites and SNPs in any given genome, the allelic variation of the marker itself is presumed to be neutral for the trait under examination. Thus, further genetic studies will be necessary to resolve the underlying cause at the *APL1* locus of this phenotypic difference.

Based on the observation that *APL1A^2^* homozygotes are less susceptible and *APL1A^1^*/*APL1A^2^* heterozygotes are more susceptible to *P. berghei* infection, one might predict that *APL1A^1^* homozygotes would in turn be exceedingly susceptible for infection. However, the G3 colony of *A. gambiae* displays a low frequency of *APL1A^1^* homozygotes (8%) and thus the sample sizes of these individuals (n = 2) obtained after a moderate number of infections was too small to include in the statistical analyses. The *APL1A^1^* homozygotes must certainly be generated each generation by the mating of heterozygotes, but due to their low frequency their fate and potential infection phenotype remains an open question. Typing mosquito developmental stages using the haplotype diagnostic showed the same haplotype frequencies in eggs and larvae as in adults (data not shown), suggesting that loss of the *APL1A^1^* homozygotes is not developmental but rather prezygotic.

## Discussion

We demonstrate by RNAi gene silencing experiments that APL1C activity is necessary to provide a high level of host-protective activity against *P. berghei* infection. By genetic association, we show that variant haplotypes at the *APL1* locus confer distinct levels of parasite susceptibility or resistance. Further functional and cell biological studies will be necessary to understand the role of *APL1C* in host defense against *P. berghei* and other parasite species, most importantly the human malaria parasite, *P. falciparum*. Further genetic studies will be required to dissect the functional basis of the distinct infection phenotypes linked to the *APL1* haplotypes.

## Methods

### Sequence Generation and Comparison

For PCR amplification and subsequent sequencing of the APL1 region, DNA was isolated from a single female G3 strain mosquito using Qiagen DNeasy (Qiagen, CA, USA). Diploid sequence was obtained via standard Sanger sequencing at the BioMedical Genomics Center at the University of Minnesota and assembled using Seqman (DnaStar, WI, USA). Due to the diploid nature of the starting DNA, no phase information is known and ambiguous base calls are given for reliably called heterozygous positions.

Three PEST clones (Clones 19600445682690, 19600445759751, 19600445654898) spanning this APL1 region were obtained from the Malaria Research and Reference Reagent Resource Center, MR4 (www.mr4.org). Clones overlapping the APL1 gene predictions were sequenced and assembled as described above (map in Figure S1C). The three assembled sequences (G3, ENSEMBL, and PEST clones) were aligned and a comparison plot drawn using ACT [22]. The score cut-off was set to 100, the % ID cutoff to 50% and the minimum size of matches was set to 100 bp or greater.

### Manual Annotation of the APL1 Gene Family

Both molecular biology and *in silico* approaches were used to manually annotate the three member genes of the APL1 family. All wet biology work was done on cDNA synthesized from total RNA isolated from a pool of 30 G3 strain female mosquitoes. Gene model predictions from a whole genome *in silico* prediction of the *A. gambiae* genome [23] were considered alongside data from 5′ and 3′ RACE reactions (First Choice RLM-RACE Kit, Ambion, Ca, USA) run with gene specific primers for *APL1A* and *APL1C* including apl1A_5′_end_R GATGGCTGTCCTCCGTTGGTACAGGC, apl1C_5′_end_R CCGTAATTTGGCTGACTTCTGTAGATT, apl1A_3′_end_F CAGCAGCAGCTCCTAGCAAGACTGCA, and apl1C_3′_end_F GGCAAGCGTTTAAGTTGCGCGAAACGCA and 5′ forward and 3′ reverse adaptor primers. The *APL1B* transcript was determined by designing primers to include all possible upstream start and downstream stop codons and screening for PCR amplification. Once the extent of transcripts was determined using this combinatorial approach, complete sequences were amplified by PCR and Sanger sequenced resulting in complete sequence of the coding regions and the ability to predict the underlying polypeptide. The resulting new gene models were submitted to Vectorbase (www.vectorbase.org) as manual annotation entries (data deposition *APL1*, manual). Three independently transcribed genes with start and stop codons are now included beginning with ENSEMBL release version 45: AGAP007036, *APL1A*; AGAP007035, *APL1B*; and AGAP007033, *APL1C*.

### Protein Structural Architecture of the APL1 Family

Predicted peptide sequences of the haplotypic forms of the APL1 family were generated from the resequencing data described above and were aligned using ClustalX [24]. SMART [25] was used to obtain predictions for the protein domains using HMMER searches and also searching for outlier homologues, Pfam domains, signal peptides, internal repeats, and regions of intrinsic protein disorder. The protein domains depicted in Figure 1B are adapted from SMART output that only displays domains more significant than established cutoffs. When two or more features occupy the same region, the following order of preference was used SMART>PFAM>PROSPERO repeats>Signal peptide>Transmembrane>Coiled coil>Unstructured regions>Low complexity.

### *APL1C* Antibodies

An *APL1C* specific gene fragment was PCR amplified with the following primers *APL1C*_F 5′ CAAGCGTTTAAGTTGCGCGAAACGCAG and *APL1C*_R 5′ CTACTTTGTAACGCGACGCGTATCTGG. The fragment was expressed from the pet-46 Ek/LIC vector (Novagen, CA, USA), and was used for production of rabbit antisera. IgG was purified from serum using Protein A IgG Purification Kit from Pierce Biotechnology (Rockford, IL, USA), and made to a concentration of 1 mg/ml. Mosquito proteins were separated by 4–12% SDS-PAGE gels of the NuPAGE Novex Bis-Tris system (Invitrogen, Carlsbad, California, USA), transferred to the Nitrocellulose membrane. Immunoblotting with the antibody against APL1C (1∶1000 dilution) was done with the Protein Detector LumiGLO Western Blotting kit (KPL, Gaithersburg, Maryland, USA). The protein loads on the blot were checked by staining with anti-ERK2 total antibody (item K-23 #sc-153, Santa Cruz Biotechnology, Santa Cruz, CA, USA) at a concentration suggested by the supplier.

### RNA Interference Assays

Gene specific fragments of *APL1 A*, *B* and *C*, *Rel*, *Cactus* and *GFP* were produced by PCR using oligos tagged at 5′end with T7 promoter sequence. The primers used were: GFP_F 5′ AGTGGAGAGGGTGAAGGTGA, GFP_R 5′ CACGTGTCTTGTAGTTCCCG, APL1A_F 5′ CTACCACCTGCCGAAAGATG, APL1A_R 5′ TCTGGTCTTGTATAGTACAATGG, APL1B_F 5′ TGAGAACAAATAAGTTCAAAGTCC, APL1B_R 5′ ACTCGCAAAGCTCAGCAAACAC, APL1C_F 5′ CCAAGAAGAACCGCAATCC and APL1C_R 5′ TCACAGTGATTTCAGGGTGTGC, REL1_F 5′GGCCCTAGTCAGCCGCAGCCG and REL1_R 5′GGGGGGTTGGAATGGATGCTT ; CACTUS_F 5′ GGTGGTGCGTCGATTGCTGG and CACTUS_R 5′ GGCTTTCGTTCAAGTTCTGTGC (all primers contained a T7 promoter sequence, 5′ TAATACGACTCACTATAGG for use in synthesizing dsRNA; the GFP fragment was used as a dsRNA control). The PCR products were used as templates for dsRNA synthesis using the MEGAscript T7 Kit (Ambion, TX, USA). Four d after the dsRNA treatment, knockdown of the target gene was verified. dsRNA treated mosquitoes were fed on mice infected with PbGFPCON (8–12% parasitaemia with mature gametocytes), a transformed strain of *P. berghei* constitutively expressing GFP [26] at 8–12% parasitaemia with mature gametocytes. Seven to 8 d following infective blood meal, midgut oocysts were counted using a florescence microscope. To compare oocyst numbers across treatments non-parametric statistical tests were used, including the Mann Whitney Rank Sum Test and the Kruskal Wallis ANOVA on ranks. For each RNAi experiment at least 30 mosquitoes survived and were counted for oocyst load. Two to 4 independent replicate infections were performed and data was pooled prior to statistical analysis.

### Indel Genotype-Phenotype Association Study

During the sequencing of the *APL1* gene family in a single G3 colony mosquito 2 segregating variants (haplotypes) were discovered (see Results). Based on available DNA sequence from homozygotes of the two segregating haplotypes, a PCR based diagnostic assay was designed to detect 2 segregating indels within *APL1A*. The first indel (46 bp in length) was located 109 bp upstream of the predicted translation start site and the second indel (237 bp in length) was located in the second exon. Amplification across this variable region results in differences in PCR product length of 191 bp in the APL1A gene, and was used to genotype mosquitoes for allelic haplotypes of the APL1 locus. Homozygous *APL1A^1^* individuals displayed a single PCR of 663 bp, homozygous *APL1A^2^* individuals displayed a single PCR band of 854 bp, and heterozygotes displayed both bands. The primers used in the haplotype diagnostic assay were 5′ GCT GGA TCC CAA CTA GTG CTG TT and 5′ AGT AAA GCA GCG GGC AGT TTG C. PCR conditions were 94°C for 1 minute, followed by 30 cycles of 94°C for 30 sec, 58°C for 30 sec, and 72°C for 45 sec and a final extension of 72°C for 7 minutes. To query for an association between haplotype and infection phenotype, G3 mosquitoes were fed on mice infected with the PbGFPCON strain of *P. berghei* (18) as described above. Seven to eight days post blood feeding midguts were dissected, oocysts counted, mosquito genomic DNA extracted from carcasses, and *APL1A* haplotype was determined by the diagnostic assay for each individual. Three replicate infections were performed and data was analyzed with a non-parametric Wilcoxon Mann Whitney test.

## Supporting Information

Figure S1 A. Reannotation of the APL1 region (reproduced from main text for clarity with Figure S1). i) Ensembl release version 36, ii) Ensembl release version 41, iii) Ensembl release version 45, iv) Empirical annotation of APL1A, B and C in this article and Vectorbase manual annotation database, v) Fragments used for RNA interference assays; common dsRNA fragment knocking down APL1A, B and C (pink), unique dsRNA fragments at the 3′ end of each gene used for gene-specific knockdowns (yellow), vi) 5′ and 3′ RACE fragments used to delimit transcripts. B. Genomic similarity. ACT Sequence Comparison plot of i) a single G3 female, ii) the genomic sequence of A. gambiae from ENSEMBL and iii) sequence from three PEST clones spanning the region. Score cut-off was set at a minimum of 100, per cent ID cut-off was set to a minimum 50% and minimum size of matches was set to 100 bp or greater. Greater sequence identity is indicated by darker shade of red. Extensive sequence similarity is evident across the APL1 locus region, with many regions >95% and most >90%. The assembled PEST strain sequence at ENSEMBL shows greater similarity to independent PEST clones than it does to G3. The LRR regions of the APL1 genes show greatest intergene similarity (diagonals). The largest region of sequence dissimilarity occurs upstream of the 5′ end of the APL1A gene. Here the ENSEMBL sequence is a string of Ns (see white box in track ii), the PEST clone has a miniature inverted transposable element (MITE) of the TA-III-Ag family based on terminal inverted repeat sequence and the G3 sequence has no MITE. C. Overlap of the PEST strain clones with the APL1 gene family. a) The APL1 gene family as presented throughout this paper, APL1A (green), APL1B (red), and ALP1C (blue) (b) The three PEST clones obtained from MR4.
(1.62 MB TIF)
