# Supplementary figures and images for: Anopheles gambiae APL1 Is a Family of Variable LRR Proteins Required for Rel1-Mediated Protection from the Malaria Parasite, Plasmodium berghei
Source: PLoS One. 2008 Nov 7;3(11):e3672. doi: 10.1371/journal.pone.0003672 (PMC2577063; doi:10.1371/journal.pone.0003672)

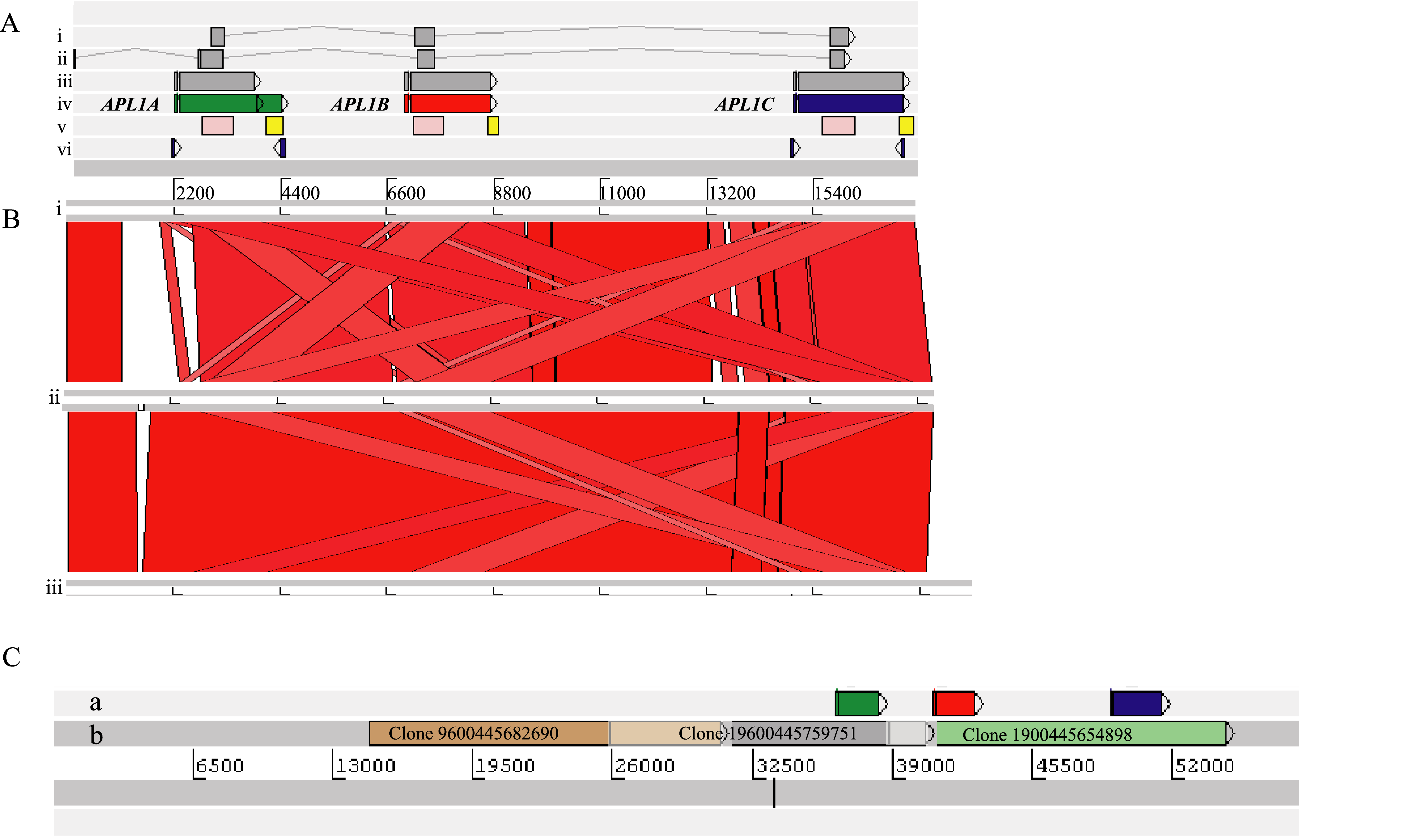

Supplement: Figure S1 — A. Reannotation of the APL1 region (reproduced from main text for clarity with Figure S1). i) Ensembl release version 36, ii) Ensembl release version 41, iii) Ensembl release version 45, iv) Empirical annotation of APL1A, B and C in this article and Vectorbase manual annotation database, v) Fragments used for RNA interference assays; common dsRNA fragment knocking down APL1A, B and C (pink), unique dsRNA fragments at the 3′ end of each gene used for gene-specific knockdowns (yellow), vi) 5′ and 3′ RACE fragments used to delimit transcripts. B. Genomic similarity. ACT Sequence Comparison plot of i) a single G3 female, ii) the genomic sequence of A. gambiae from ENSEMBL and iii) sequence from three PEST clones spanning the region. Score cut-off was set at a minimum of 100, per cent ID cut-off was set to a minimum 50% and minimum size of matches was set to 100 bp or greater. Greater sequence identity is indicated by darker shade of red. Extensive sequence similarity is evident across the APL1 locus region, with many regions >95% and most >90%. The assembled PEST strain sequence at ENSEMBL shows greater similarity to independent PEST clones than it does to G3. The LRR regions of the APL1 genes show greatest intergene similarity (diagonals). The largest region of sequence dissimilarity occurs upstream of the 5′ end of the APL1A gene. Here the ENSEMBL sequence is a string of Ns (see white box in track ii), the PEST clone has a miniature inverted transposable element (MITE) of the TA-III-Ag family based on terminal inverted repeat sequence and the G3 sequence has no MITE. C. Overlap of the PEST strain clones with the APL1 gene family. a) The APL1 gene family as presented throughout this paper, APL1A (green), APL1B (red), and ALP1C (blue) (b) The three PEST clones obtained from MR4. (1.62 MB TIF) [file pone.0003672.s001.tif]
